# Hypoxic and Thermal Stress: Many Ways Leading to the NOS/NO System in the Fish Heart

**DOI:** 10.3390/antiox10091401

**Published:** 2021-08-31

**Authors:** Mariacristina Filice, Sandra Imbrogno, Alfonsina Gattuso, Maria Carmela Cerra

**Affiliations:** Department of Biology, Ecology and Earth Sciences, University of Calabria, 87036 Arcavacata di Rende, Italy; mariacristina.filice@unical.it (M.F.); maria_carmela.cerra@unical.it (M.C.C.)

**Keywords:** nitrergic system, fish heart, cardiac performance, multiple stress, hypoxia, thermal changes, AMPK

## Abstract

Teleost fish are often regarded with interest for the remarkable ability of several species to tolerate even dramatic stresses, either internal or external, as in the case of fluctuations in O_2_ availability and temperature regimes. These events are naturally experienced by many fish species under different time scales, but they are now exacerbated by growing environmental changes. This further challenges the intrinsic ability of animals to cope with stress. The heart is crucial for the stress response, since a proper modulation of the cardiac function allows blood perfusion to the whole organism, particularly to respiratory organs and the brain. In cardiac cells, key signalling pathways are activated for maintaining molecular equilibrium, thus improving stress tolerance. In fish, the nitric oxide synthase (NOS)/nitric oxide (NO) system is fundamental for modulating the basal cardiac performance and is involved in the control of many adaptive responses to stress, including those related to variations in O_2_ and thermal regimes. In this review, we aim to illustrate, by integrating the classic and novel literature, the current knowledge on the NOS/NO system as a crucial component of the cardiac molecular mechanisms that sustain stress tolerance and adaptation, thus providing some species, such as tolerant cyprinids, with a high resistance to stress.

## 1. Introduction

Living organisms are constantly exposed to stress. After Seyle’s definition of stress as “the non-specific response of the body to any demand placed upon it” and the consequent declinations of the different degrees of responses from eustress to distress [1,2], decades of research have established that the ability to face stress-dependent challenges represents a basic mechanism to maintain organism homeostasis. Many factors shape the stress response, making the scenario highly complex. In the attempt to draw a framework in which networks and circuits involved in the crosstalk between the environment and the organisms are recapitulated, several stress-related concepts have been developed, such as *stressome* (the catalogue of genes and their products involved in maladaptive stress response) and *stressotope* (the adaptive background that includes the circuits of molecular mediators involved in the stress response, up to the population level) [3]. An important aspect in the context of the adaptive response is that multiple stresses often converge in challenging the organism, and this elicits a response that is the result of many events, occurring at a multilevel scale, from the whole organism to molecular signalling [3]. This is the case of related stresses, such as hypoxia and temperature that, particularly in water environments, move together. Temperature variations inversely affect water O_2_ solubility; at the same time, thermal modifications impact on the energy demand of aquatic ectothermic species. Both O_2_ and temperature dynamically change over a period of days, seasons, years, and even on a geographic scale for migratory species, so that animals are constantly and physiologically exposed to the requirement of adaptation [4,5]. In recent times, the stress induced by changes in water O_2_ and temperature is severely increasing due to the intense human manipulation of the environment and to the addition of other factors, for example non-natural chemical species (see for reference [6]). This further challenges the intrinsic tolerance of the animal.

Under stress challenges, either single or multiple, the survival of the organism is allowed by the adaptive flexibility of crucial organs such as the brain and the heart, of which the fundamental functions need to be strictly preserved. Accordingly, these organs are regarded with interest as bioassays in studies related to stress adaptation.

An important body of the literature describes the mechanisms that, in aquatic species, sustain cardiac adaptation to environmental challenges, with attention not only to the upper and lower limits of this adaptation, but also to the molecular pathways that are recruited during the exposure to multiple challenges, an event that is becoming more and more frequent in damaged natural environments. A recent example is the cardiac transcriptomic response described in *Fundulus grandis* developing larvae to four combined stressors (O_2_ availability, temperature, salinity, and polycyclic aromatic hydrocarbons) [7]. It was found that each single stress, administered alone, affects the heart in terms of beat-to-beat hemodynamic and development. At the same time, stress combination potentiates the effects on the heart, either positively or negatively, by affecting canonical pathways involved in heart contractility, vasomotility, and cardiomyocyte proliferation. These pathways include the cardiac nitrergic system [7]. As demonstrated by many papers, in the heart, this system is at the crossroads of many stress-sensitive circuits [8,9,10,11,12,13]. It is under a modulation elicited by stress, and at the same time, it is crucial for shaping the stress response, either adaptive or maladaptive.

**Figure 1:**
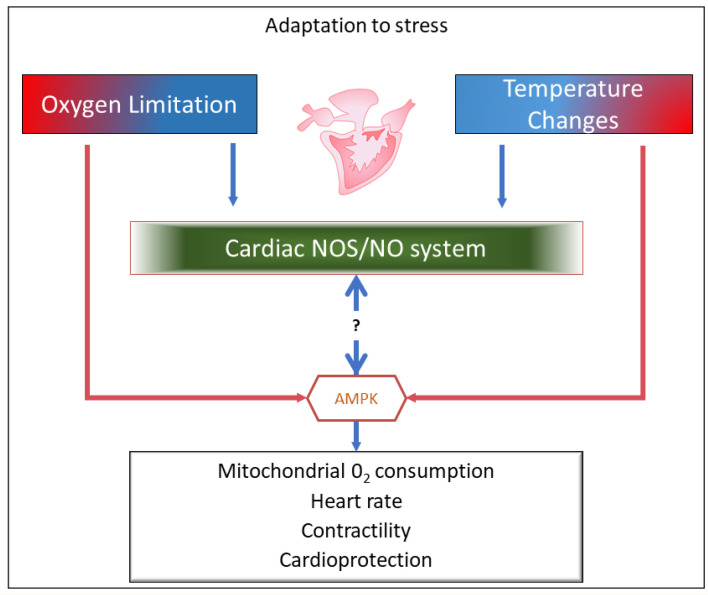
Graphical representation of the review content. Under conditions of O_2_ limitations and temperature variations, the nitric oxide synthase (NOS)/nitric oxide (NO) system, by directly or indirectly interacting with critical kinases, e.g., AMP-activated protein kinase (AMPK), modulates molecular mechanisms that in the fish heart sustain stress tolerance and adaptation.

The following paragraphs will focus on cardiac physiological responses of fish to O_2_ limitations and temperature variations, used as examples of the large variety of challenges that animals are naturally required to face. In this context, the nitric oxide synthase (NOS)/nitric oxide (NO) system will be discussed as a crucial component of the molecular mechanisms that shape the stress response of the fish heart, particularly in species characterized by a high adaptive flexibility, as in the case of cyprinids (Figure 1). A preliminary description of the NOS/NO system in the control of basal cardiac function and essential information on the cardiac response to O_2_ and temperature changes in teleost fish will be also provided for unfamiliar readers, since this information is the starting point for all evaluations related to the involvement of the nitrergic system in the stress response.

## 2. The NOS/NO System and the Fish Heart

NO is a gasotransmitter generated by the family of NOS isoenzymes, which include the constitutive endothelial (eNOS) and neuronal (nNOS) and the inducible (iNOS) isoforms. By using molecular O_2_ and NADPH as essential cofactors, these enzymes convert L-arginine into L-citrulline and NO.

NO exerts its physiological effects either by soluble guanylate cyclase (sGC)-dependent mechanisms or reacting with hemes, thiols, or amines, forming iron-nitrosyl (FeNO), S-nitroso (SNO), and N-nitroso (NNO) compounds [14]. NO has a very short half-life. It is rapidly metabolized to nitrite in reaction with O_2_ [15] and is inactivated by oxidation to nitrate in reaction with oxygenated hemoglobin (Hb) and myoglobin (Mb). Under hypoxic and/or acidic conditions, nitrite may represent a reservoir of NO, since it can be reduced back to NO by a variety of non-enzymatic and enzymatic pathways. These include acidic disproportionation and reaction with endogenous nitrite reductases, such as deoxygenated Hb, Mb, neuroglobin, cytoglobin, xanthine oxidoreductase, eNOS, and some mitochondrial enzymes [16].

The heart is a major NO producer and, at the same time, a target of its actions. Generated by myocardial and non-myocardial tissues, NO elicits an autocrine–paracrine control of beat-to-beat, short-term, and long-term cardiac responses [17,18].

Although non-univocal results can be found in the literature, it is currently recognized that, as in mammals, a functional NOS/NO system is present in the fish heart [19,20,21,22]. nNOS and iNOS have been identified in teleosts. Moreover, physio-pharmacological and immunodetection approaches indicate the presence of an “eNOS-like” isoform in the heart of several species. The presence of a canonical eNOS in fish remains questioned. However, Andreakis and coworkers [23] suggested that an nNOS isoform, with an endothelial-like consensus, may cover some functional features typical of the eNOS isoform. As an example, a myristoylation consensus sequence in nNOS from the lamprey and in iNOS from the zebrafish is suggested to perform functions comparable to the mammalian eNOS [24].

Cardiac NOSs have been documented in eurythermal fish species (*Thunnus thynnus thynnus* [25]; *Anguilla anguilla* (*A. anguilla*) [25,26]; *Carassius auratus* (*C. auratus*) [11]), cold-adapted Antarctic teleosts (icefish *Chaenocephalus aceratus* (*C. aceratus*), hemoglobinless *Chionodraco hamatus* (*C. hamatus*), and red-blooded *Trematomus bernacchii* (*T. bernacchii*) [27,28]), and lungfish (*Protopterus dolloi* [29] and *Protopterus annectens* [10]). In these species, the enzyme is mainly expressed in the endocardial endothelium (EE), indicating this tissue as a relevant paracrine source of NO. It is also present, to a lesser extent, in myocardiocytes that may act as an additive autocrine NO source. Interestingly, NOSs are also detected in the ventricular visceral pericardium (i.e., epicardium), suggesting that this tissue is a source of nitrergic signalling. This extensive intracardiac expression of NOSs isoforms has been considered indicative of the ability of NO to interact with a number of cellular targets to achieve a spatio-temporal modulation of the cardiac function in short- and long-term responses, under basal conditions and in the presence of physical and chemical stimulation [21,30].

A basal nitrergic control has been documented in the heart of different fish species. In steelhead trout (*Oncorhynchus mykiss* (*O. mykiss*)), exogenous NO administration enhances myocardial relaxation rates and this, in turn, influences isometric twitch duration and muscle contractility. This NO-dependent effect is related to contraction frequency, as the twitch duration is reduced by 25% at a frequency of 20 beats per min (bpm), but only by 5% at 80 bpm [31]. In eel [32], salmon [33], and goldfish [11], a tonic release of autocrine NO negatively modulates the basal mechanical performance of the heart. In addition, NO influences the Frank–Starling mechanism (salmon [33]; eel [34]; goldfish [11]), a fundamental cardiac trait that, in all vertebrates, allows the myocardium to increase stroke volume (SV), and consequent cardiac output (CO), in response to increased venous return (preload). As detected in the goldfish isolated and perfused heart, the sensitivity to filling pressure decreases, when NO generation is prevented by NOS inhibition via L-NMMA or, downstream, when cGMP production is blocked by ODQ [11], thus involving the classic NO/cGMP-dependent cascade. Like the recent data in the steelhead trout by Carnevale and colleagues [31], in the eel, the nitrergic control of the Frank-Starling response is related to an enhanced relaxation, possibly due to calcium reuptake by SERCA2a pumps controlled by phospholamban S-nitrosylation [28].

In addition, in the diseased fish heart, NO influences the myocardial response to stretch. This was observed in Atlantic salmon (*Salmo salar* (*S. salar*)) affected by ISA (infectious salmon anemia), in which the heart is characterized by an impaired performance correlated with the severity of the pathology. In diseased fish, cardiac functional damage is reversed, when the NO availability for the tissue is reduced by selective iNOS inhibition. In contrast, no effects are observed in healthy animals under NO deprivation. This suggested that, in the presence of infections, NO derived by iNOS induction may be mechanistically linked to the cardiac fragility consequent to the disease [33].

The membrane permeable nature of NO allows it to freely diffuse and control adjacent cells. In the avascular, or poorly vascularized, fish heart, the EE represents the major source of NO. The data from our laboratory reveal that this EE-derived NO is obligatory for the contractile effects elicited by many endoluminal chemical stimuli such as acetylcholine, angiotensin II, vasostatin-1, and β3-adrenoreceptor [32,35,36,37].

Of note, NO produced in the heart can mediate actions distal from the site of production thanks to the storage into the blood as nitrite. In fish, compared to terrestrial animals, the nitrite pool can be enriched by uptaking nitrite from the environment across the respiratory surfaces [16]. In addition, as observed in the crucian carp (*Carassius carassius* (*C. carassius*)), the tissue nitrite pool may be further increased by the nitrate reductase activity generally mediated by xanthine oxidoreductase and Mb [38]. This results in an enhanced NO availability with consequent larger effects on the cardiovascular function. This mechanism is of particular importance during deep hypoxia and anoxia when, being NOS enzymes unable to produce NO because of the absence of O_2_, it contributes to cardioprotection [38,39]. Data in the zebrafish support this possibility showing that exposure to water NO_2_ is accompanied by a significant NO production, together with vasodilation, and a decrease in blood pressure [40]. In the presence of environmental hypoxia, these events may be amplified by stimulating the reduction of nitrite to NO (see [11] for references).

The role of NO_2_ in the cardiac nitrergic control has been studied by Cerra and colleagues in the eel *A. anguilla*, and in the icefish *C. hamatus*, two species characterized by extremely different ecophysiological traits [41]. The eel is a eurytherm and euryhaline teleost that experiences considerable fluctuations in environmental O_2_. In contrast, the icefish is an extreme stenotherm, endemic to the stable, icy, and richly oxygenated Antarctic water. In the eel, nitrite negatively influences the contractility of the isolated and perfused heart. This occurs by modulating the NOS activity and the cGMP/PKG pathway. Moreover, nitrite influences the Frank–Starling response through a mechanism which recruits the NO/cGMP/PKG pathway and requires protein S-nitrosylation [42]. Contrary to the eel, in the icefish nitrite induces a NOS-dependent increase in contractility, similar to the effect induced by NO. Icefish lacks Hb, a key protein in NO homeostasis, which is able to not only scavenge NO, but also generate it from NO_2_ (for references, see [41]). It has been proposed that, in the icefish, the reduction of NO_2_ to NO occurs through cardiac Mb that in this fish may represent the predominant form of NO_2_ reductase [41].

### 2.1. Hypoxia

Water hypoxia (O_2_ levels: ≤2.8 mg L^−1^; [43]) is a major limiting environmental factor. It is routinely experienced by aquatic animals, either chronically or on a diel or seasonal basis, and results from complex processes including mixing, air–water exchange, and fluctuations in the pattern of O_2_ production and consumption [44,45]. Hypoxia is common in areas characterized by low mixing or light limitation (e.g., densely vegetated swamps, flooded forests, and deep waters), as well as in tropical freshwaters, where high temperatures increase organic decomposition, thus reducing O_2_ solubility [46]. In recent years, aquatic hypoxia is increasing due to anthropogenic influences and climate changes [47,48], with severe impact on individual organisms, communities, and ecosystems. The presence of specialized branchial neuroepithelial cells (NECs) allows teleosts to “sense” oxygen changes in water/blood milieu [49]. Fish exhibit a very large spectrum of O_2_ sensitivity moving from species that dramatically suffer O_2_ deprivation to species which are able to tolerate hypoxic or even anoxic environments. For example, salmon and tuna extensively rely on aerobic metabolism for rapid and sustained swimming, being extremely sensitive to hypoxia [50,51], while the eel and the hagfish perform well also at low O_2_ levels [52,53]. Members of cyprinids, such as the goldfish (*C. auratus*) and the crucian carp (*C. carassius*) are among the most hypoxia tolerant vertebrates, being able to survive O_2_ reduction or even deprivation for long periods [54]. A variety of specializations contribute to dealing with hypoxia; these include behavioral, physiological, biochemical, and molecular responses, which allow either enhancing O_2_ uptake from the environment or limiting potentially damaging consequences to cells, tissues, and organs [54,55].

In the heart, a general response to hypoxia is bradycardia, together with the depression of myocardial contractility, and the O_2_ consumption rate. These effects are particularly evident in hypoxia-intolerant species [56,57,58]. In contrast, hypoxia-tolerant species such as the crucian carp and the goldfish retain a normal cardiac function and even potentiate it by activating a complex molecular machinery that is only partially known [11,59,60]. As observed on ex vivo isolated and working cardiac preparations of the goldfish, the exposure to acute hypoxia is accompanied by an increased myocardial sensitivity to preload increases (i.e., the Frank–Starling mechanism) [11].

The hemodynamic changes which occur under low oxygen conditions are considered crucial for maintaining functional and metabolic interaction between organs and tissues, allowing hypoxia tolerance to the whole organism [11]. When exposed to low O_2_ levels, the goldfish relies on great liver and muscle glycogen reserves, reduces metabolism and avoids lactic acidosis by converting lactate to ethanol and CO_2_, which is rapidly eliminated through the gills [54,61,62]. The preserved or even increased cardiovascular function not only contributes to mobilizing glucose from the hepatic glycogen store, but also allows the transport of lactate to the muscle for its conversion to ethanol [59].

O_2_ delivery to the heart is mandatory for maintaining heart performance [63,64]. In fish, different types of blood supply and cardiac architecture are present. On a species-specific base, the ventricle may be exclusively composed of a spongy myocardium perfused by poorly oxygenated venous lacunary blood, or it may include an outer layer of compact tissue, of different thickness, perfused by O_2_-rich blood from the coronary circulation (for references and details on the structural characteristics of the fish heart see [65,66,67]). In the venous blood reaching the *spongiosa*, O_2_ saturation can decrease from about 25% under normoxia to about 3% during hypoxia [68,69,70]. Thus, the spongy myocardium may be exposed by very low O_2_, and this requires a specific tissue adaptation. In fact, a low sensitivity to hypoxia was demonstrated in the *spongiosa* of the steelhead trout (*O. mykiss*) with respect to the compact myocardium [71].

### 2.2. The NOS/NO System in the Fish Heart under Hypoxia

A general effect of the activation of the NOS/NO system in the heart under hypoxia is the limitation of mitochondrial O_2_ consumption for the NO competitive binding with O_2_ to CytC oxidase [72]. When O_2_ availability is reduced, this contributes to preserving myocardial efficiency by enhancing the force generated per O_2_ consumed [73,74].

A comparison between the cardiac response to low O_2_ in hypoxia-intolerant vs. hypoxia-tolerant fish has been carried out by Pedersen and collaborators by using ventricular strips from trout and goldfish [57]. In both species, NO generated by NOS activation inhibits respiration rates and contributes to improving myocardial efficiency. However, when NO is generated from nitrite conversion, different behaviors are observed in the two species. In fact, in trout but not in goldfish, myocardial O_2_ consumption is reduced without changes in force development. This is attributed to differences in oxygen affinity and then in the nitrite reductase capacity of myocardial Mb. With less O_2_ available, trout Mb may readily de-oxygenate, thus generating NO from nitrite, while the goldfish Mb, by remaining saturated with O_2_, is prevented by reducing nitrite [57].

In the steelhead trout, the role of the nitrergic system in the mechanical response of the heart to low O_2_ has been further investigated by Carnevale and colleagues [31]. By exposing spongy ventricular strips from animals acclimated to low oxygen (PO_2_ = 8 kPa) to the NO donor (SNP), they observed that hypoxic acclimation scarcely influences the frequency-related NO-dependent effect on twitch duration and muscle contractility. The above studies suggest that, in trout, hypoxia exposure does not significantly influence the cardiac isometric contractility in response to NO. However, the authors do not exclude the possibility that the use of muscle strips may fail to reveal additional effects of NO that may be preserved in the whole heart preparation, closer to the in vivo situation [31,57]. In line with this, evidence obtained in goldfish, by using ex vivo isolated and perfused working heart preparations, a technique that prevents the constraints imposed by the use of limited parts of the organ, shows that the potentiated basal performance, typical of acute O_2_ limitation, is accompanied by an increased myocardial NOS expression [11]. The possibility that a more expressed enzyme generates a higher amount of NO and this in turn affects the myocardial performance is confirmed by the evidence that NO scavenging with PTIO, as well as NOS inhibition by L-NMMA, reduces the hypoxia-dependent increase of contractility. Moreover, under hypoxia, NOS inhibition by L-NMMA unchanges the Frank–Starling response of the goldfish heart. In contrast, a significant reduction of the myocardial sensitivity to stretch is observed, if NO is removed from the tissue by PTIO and if sGC is inhibited by ODQ. This is of relevance, since it indicates that the effects elicited by NO involve the cGMP cascade but are NOS-independent, thus requiring other routes for NO generation [75].

The molecular mechanisms that allow NO to play a role in the myocardial response to hypoxia have been only partially defined in fish. In the mammalian heart, under basal conditions and in the presence of low O_2_, the PI3-K/Akt pathway controls eNOS activity, and thus NO generation and the consequent NO-dependent signalling, being protective for the heart [76,77]. The same occurs in hypoxia-tolerant fish in which, as observed in the perfused goldfish heart, exposure to low O_2_ is accompanied by the activation of these kinases [75]. Interestingly, in the hypoxic goldfish heart, the molecular events of downstream NO generation exclude the involvement of the cGMP-dependent signalling [75]. Non-cGMP-dependent pathways represent an important route for NO to control its molecular targets. These pathways are mainly represented by protein S-nitrosylation, the covalent attachment of NO to the thiol group of cysteine (Cys) residues [78]. A significant reduction in the degree of S-nitrosylated proteins has been reported in the hypoxic goldfish heart with respect to the normoxic counterpart. In mammals, the significance of a dysregulated protein S-nitrosylation is correlated with both cardiac disorders [79] and with protective mechanisms against the development of myocardial dysfunction under stress [80]. Proteins encountering denitrosylation in the hypoxic goldfish heart and the related functional significance have not yet been identified. However, it is reasonable to hypothesize that this event, by activating still undefined protective programs, contributes to preserving myocardial function when challenged by hypoxia [75].

It is known that, under hypoxia, NO may determine protein nitration. This consists in the substitution, mainly under the action of peroxynitrite (ONOO^–^), of a nitro group to tyrosine residues, to give 3-nitrotyrosine [81]. Nitration is generally associated with alterations of protein catalysis, protein–protein interaction, and tyrosine kinase signaling [82]. However, a nitration-dependent control of redox homeostasis is also present in normally functioning cardiac muscle [83]. Interestingly, data obtained in the hypoxic goldfish heart suggest the presence of hypoxia-induced nitration, since an increased expression of Nox2, the catalytic subunit of NADPH oxidase [75], and 3-nitrotyrosine [84] has been reported. If further data confirm the occurrence of nitration, putative targets must be identified. Based on the available information, some proteins can be hypothesized. One is the SERCA2a pump, the integral membrane protein controlling cardiac Ca^2+^ homeostasis by actively transporting the ion into the sarcoplasmic reticulum. It is susceptible to nitrosative and oxidative modifications for the presence of several cysteine and tyrosine residues [83,85]. The structural proximity to mitochondria exposes SERCA2a pumps to reactive O_2_/nitrogen species generated as by-products of the oxidative phosphorylation [86]. The nitrotyrosine modification of SERCA2a has been observed in several pathophysiological conditions [87], and nitrated SERCA2a is utilized as a cardiac marker of nitrative stress [83]. Although direct evidence on SERCA2a pumps nitration in fish is not available, the significant reduction of the hypoxia-induced time-course increase of the goldfish heart performance observed under conditions of SERCA2a inhibition [75] points to SERCA2a as a putative target of nitration in the hypoxic heart.

In general, it appears from the available information that, in the goldfish heart, NO activates a protective program that sustains the performance under hypoxic challenge. Consistent with this, it was found that NO positively modulates cardiac sarcolemmal KATP channels, a response that, like the KATP-dependent protection observed in the ischemic mammalian myocardium [88], may contribute to the cardiac hypoxia tolerance of this species [8]. In addition, the hypoxic goldfish heart also shows an enhanced expression of the hypoxia inducible factor α (HIF-1 α) [11]. In mammals, HIF1α/NO interaction is involved in hypoxia-elicited cardio-protective responses. Under hypoxia, HIF-1α activates genes critical for cell survival, including NOS [89,90,91]; at the same time, high NO concentrations (>1 μM) stabilize HIF-1α, leading to an increase in the dimeric form of protein which, by binding HREs sites, and enhances NOS gene expression and thus NO generation [92].

### 2.3. Temperature

Aquatic ectotherms depend on the thermal milieu to regulate their metabolic rate. Thermal tolerance is limited by the capacity to meet adequate O_2_ demands of tissues [93,94,95,96]. Apart from species living in extremely stable environments, many fish routinely face temperature fluctuations associated not only with ontogenetic and/or seasonal changes, but also with diurnal changes especially in shallow water bodies. Nevertheless, their phenotypic plasticity (developmental or reversible acclimation) allows compensation by altering tolerance limits for optimizing the performance under changed temperature regimes [97,98,99,100,101]. While eurythermal fish, naturally subjected to large temperature changes, develop acclimation strategies for preserving their fitness, stenothermal species show specific evolutionary adaptations at the expense of reduced plasticity.

In many eurytherm fish, temperature changes importantly influence the cardiac function that requires to be modulated to ensure an adequate CO. In addition, the upper thermal tolerance is partly determined by the capacity of the heart to ensure an adequate systemic O_2_ delivery [102]. This occurs by changing the heart rate (HR) more than the stroke volume [103,104]. When temperature acutely rises, the HR increases before declining at temperatures preceding the critical thermal maximum [103,104] and this compromises the cardiac function [102,105,106]. On the other hand, when the temperature drops, bradycardia occurs [107], and this is associated with an increased diastolic duration to maintain CO by increasing filling time and with little modifications of the systolic duration [108]. Moreover, prolonged temperature variations induce a remodelling of the cardiac function, but the effects may be quite different from those observed under acute changes ([107] and references therein). In fact, while acute rise in temperature typically increases the rate of pacemaker potential, a longer exposure to warmer temperature decreases the intrinsic HR [109,110]. The resetting of the pacemaker rate may occur over different time scales and may depend on different population, species, and experimental conditions [110,111,112]. In addition, myocardial excitability is influenced by temperature. At critically high temperatures, this has been ascribed to an imbalance of inward *I*_Na_ and outward *I*_K1_ currents [113,114]. Changes in myocardial electrical properties induce a functional atrioventricular block with a consequent bradycardia limited to the ventricle which becomes unable to follow the sinoatrial rate [114].

Cardiac remodelling accompanies the response of the fish heart to temperature acclimation [107,115,116,117]. However, large intra- and inter-specific differences prevent a general picture describing the cardiac response to either warm/cold acute exposure or acclimation to temperature challenges. A reduced ventricular mass is observed under warm acclimation with a consequent impairment of the stroke volume and without changes in the CO which is maintained through an improved contractility and quicker rates of ventricular contraction and relaxation [115]. Conversely, cold acclimation induces cardiac hypertrophy. In this case, a larger ventricular muscle mass compensates for the decreased contractility, maintaining the stroke volume and thus CO at lower temperatures [107,115,116].

Different from eurythermal species, stenotherm fish scarcely tolerate thermal challenges. This is the case of Antarctic teleost Channichthyidae that live in the extremely stable, frigid, and highly oxygenated Antarctic waters [118,119]. Some of them are unique among adult vertebrates, since they lack hemoglobin (Hb; [120]) and, in some species, also Mb [121]. This is compensated by extensive cardiocirculatory remodelling such as hypervolemia, low blood viscosity, large capillaries, cardiomegaly, and high blood flow with low systemic pressure and systemic resistance ([119] and references therein). It is possible that the absence of respiratory pigments may contribute a modest thermal plasticity, making these teleosts vulnerable to short-term extreme temperature fluctuations, as well as long-term climate warming [122,123]. However, recent comparative studies performed in several Antarctic teleosts with and without respiratory pigments suggest some degree of cardiac accommodation to temperature variations, particularly in species expressing both Hb and Mb [124].

### 2.4. The NOS/NO System in the Fish Heart under Temperature Challenges

In fish, the NOS/NO system plays a role in the regulation of the cardiac function in species adapted to both temperate and extreme thermal regimes, as well as in animals differently tolerant to thermal stress.

In the eurythermal eel *A. anguilla*, the NOS/NO-dependent modulation of the Frank–Starling response [32,34] is impaired by temperature changes [125]. Amelio and coworkers observed that the positive modulation elicited by the intracardiac NO release on the Frank–Starling response disappears when animals are acutely exposed at temperatures lower or higher than the acclimation one, both in the case of spring- and winter-like (acclimation temperature: 20 °C and 10 °C, respectively) conditions. These effects are paralleled by reduced expression levels of NOS and pAkt, suggesting that the NO production via the Akt/NOS axis is temperature-dependent [125]. The authors propose that the abolition of the nitrergic modulation of the Frank–Starling response under thermal challenges may involve protein kinases, suggesting Akt as an temperature-sensitive element in the NO-generating transduction cascade [125].

Another example is provided by salmonids. In the eurythermal Atlantic salmon, long-term exposure to temperature enhancement is accompanied by an increased expression of iNOS in both compact and spongy ventricular myocardium, indicative of an enhanced NO production. At the same time, also VEGF expression increases [126]. This is interesting, since the two effects, if considered together, may call for a potentiated blood supply to the myocardium obtained by increasing vascularization (via VEGF) and/or by dilating the vessels (via NO). In fact, in salmonids, NO is known to induce vasodilation and reduce coronary resistance [19], thus contributing to compensating for the increased O_2_ demand under elevated temperature.

The relationship between temperature variations and the cardiac nitrergic control may be of great importance in fish living under extreme temperatures. Unfortunately, this aspect remains unexplored, although the information so far available indicates that the NOS/NO system plays a role in the modulation of the basal cardiac performance of these animals.

In Antarctic teleosts, functional NOSs are present in the heart of the hemoglobinless *C. aceratus* and *C. hamatus* and the red blooded *T. bernacchii.* An eNOS-like enzyme is mainly present in the lacunae of the spongy ventricle, while iNOS is basally expressed in the cytoplasm of myocardiocytes [27,28]. Despite the similar distribution, physio-pharmacological studies show that NO differently affects the contractility of the three species [28]. In fact, endogenous NO (L-arginine administration) reduces contractility in *T. bernacchii*, contrary to the stimulatory effect observed in the two icefish species. In addition, while in *C. hamatus* the NO-induced effects are cGMP-dependent, in *T. bernacchii* and *C. aceratus* these effects are cGMP-independent. The authors suggest that, in the absence of respiratory pigments, the loss of NO-oxygenase activities associates with Hb/Mb and the consequent increased NO levels may account for the observed differences [28]. In addition, the higher NO amount in the icefish may be of relevance for counterbalancing the adrenergic stimulation of the in vivo contractility that is part of the cardiac thermal response of these teleosts [124]. Further studies are welcome to understand whether the absence of respiratory pigments, associated with an increased cardiac NO availability, may be beneficial or detrimental for the resilience of these fishes to prolonged temperature challenges.

Tropical lungfish represent a peculiar model organism, since during warm seasons they undergo aestivation, a metabolic adaptation associated with functional modifications in tissues and organs including heart, kidney, gills, lung, and skeletal muscle [10,29,127,128,129]. A very interesting aspect of the lungfish is the ability of the myocardium to ensure contractility during warm aestivation, maintaining an appropriate blood perfusion to the whole organism. The lungfish heart, as observed in *Protopterus dolloi* [29] and *Protopterus annectens* [10], expresses NOS enzymes, and this expression increases under aestivation [29]. It has been proposed that the consequent enhanced NO release preserves the heart by sustaining cardiac bioenergetics in the presence of metabolic depression and reduced myocardial O_2_ consumption [10,29,128].

### 2.5. Single Stress to Multiple Stress: There Is Room for the Nitrergic System in the Fish Heart

Cardiac function is constantly challenged by the convergence of multiple stresses. In animals living in the water milieu, O_2_ limitations and temperature changes are extremely frequent. However, the cross-interaction between these two variables in the heart has been scarcely explored.

In fish, O_2_ availability is generally believed to be a major determinant of the thermal tolerance, with exceptions possibly due to specific adaptations and/or to the role of additional internal and/or external constraints [130]. Very recently, experiments carried out on the instrumented in situ heart of the hypoxia-tolerant sablefish (*Anoplopoma fimbria* (*A. fimbria*)) attempted to analyze the cross-tolerance between the two related events of O_2_ limitation and increased temperature. The results reveal that preliminary exposure to low O_2_ followed by warming makes the heart unable to increase the HR and the CO. The authors suggest a role for the nervous system and hypothesize that hypoxia may severely limit the cardiac response to thermal challenges [96].

In addition, the role elicited by the NOS/NO system, when the fish heart is challenged by the simultaneous exposure to temperature and O_2_ variations, has received little attention, so that, at the moment, any conclusion can be only inferential. It has been shown by functional data on trout (*O. mykiss)* ventricular strips that the beneficial effects induced by NO on the cardiac performance under hypoxia are reduced, or even reversed, if the HR is increased [31]. Interestingly, as illustrated above, acceleration is the typical response of the fish heart to thermal increases, being crucial for matching the pump function to the organism demand [103,131]. Accordingly, it can be assumed that, if the fish is exposed to both temperature rise and O_2_ limitation, the enhanced HR may aggravate the consequences on the contractility of the loss of NO-dependent protection.

At the subcellular level, both temperature changes and hypoxia converge on mitochondria that are thermal and O_2_-sensitive. This is important for fish that, being ectotherms, need to optimize aerobic metabolism when temperature changes [132,133]. At temperatures acutely incremented to the thermal limit, fish cardiac mitochondrial function is altered in terms of proton leakage rates, oxidative phosphorylation, membranes integrity, protein complexes function, and oxidative imbalance due to increased ROS production [132,134,135,136]. However, as reported in the Atlantic salmon (*S. salar*), warming acclimation (20 °C, close to the upper thermal tolerance of the species) mitigates the impairment of cardiac mitochondrial function, suggesting a plasticity that can be beneficial for thermal tolerance. In addition, warming acclimation decreases the NO-dependent inhibition of cardiac mitochondria function [137,138]. This may better comply with the increased metabolic demands associated with heat stress, although it is unknown whether this is beneficial during acclimation to temperatures close to the upper thermal tolerance [138].

Few studies explored, in fish, the influence of O_2_ limitation on mitochondrial functions and the consequences on cardiac performance. Conflicting evidence has been obtained, possibly depending on experimental conditions and/or the species-specific resistance to hypoxia [139,140]. Similarly, the role of the nitrergic system has been almost ignored, with the exception of the data obtained in the sablefish *A. fimbria*, in which the effects of NO on mitochondrial respiration have been investigated together with the mitochondrial sensitivity to NO in relation to O_2_ availability [141]. It was found that the long-term exposure of the animals to hypoxia increases cardiac mitochondrial sensitivity to NO inhibition. However, as also discussed by the authors, it is unclear whether this may be considered a general response or a peculiar trait of this species that is characterized by a high hypoxia tolerance [141]. To the best of our knowledge, no information is available concerning the involvement of the nitrergic system in modulating the cardiac mitochondrial function of fish when thermal increments and hypoxia converge on the animals.

**Figure 2:**
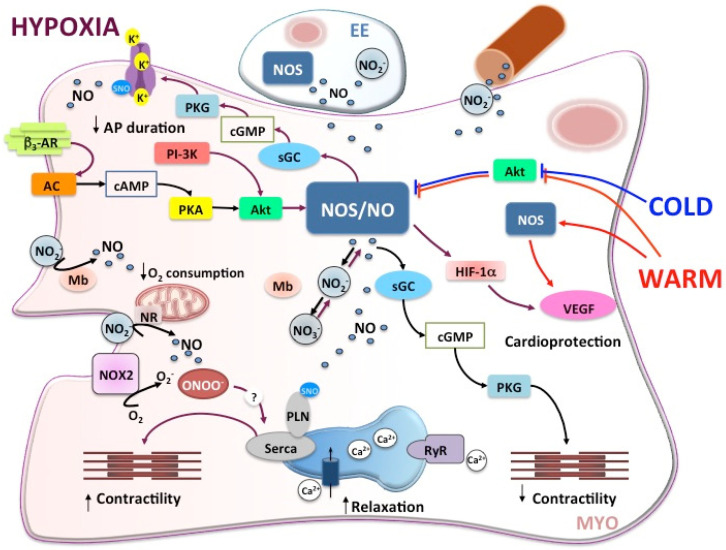
Schematic overview of the NOS/NO-mediated intracellular pathways activated in fish cardiomyocyte (MYO) under basal (black arrows), and hypoxic (violet arrows), and thermal stress (blue and red arrows). For details, see the text. AC, adenlylate cyclase; AKT, serine/threonine kinase 1; β3-AR, beta 3 adrenoreceptor; cAMP, cyclic adenosine monophosphate; cGMP, cyclic guanosine monophosphate; EE, endocardial endothelium; HIF-1α, hypoxia inducible factor-1α; Mb, myoglobin; NO, nitric oxide; NOS, nitric oxide synthase; NO_2_^–^, nitrite; NOX2, NADPH-oxidase; NR, nitrite reductase; ONOO^–^, peroxynitrite; PI-3K, phosphatidylinositol 3-kinase; PKA, protein kinase A; PKG, protein kinase G; PLB, phospholamban; RyR, ryanodine receptor; SERCA, sarco-endoplasmic reticulum Ca^2+^-ATPase pump; sGC, soluble guanylyl cyclase; SNO, S-nitrosylation; SR, sarcoplasmic reticulum; VEGF, vascular endothelial growth factor.

NOS/NO pathways activated in the fish heart in response to hypoxia and temperature stress are summarized in Figure 2.

## 3. Upstream and Downstream the NOS/NO System: AMP-Activated Protein Kinase (AMPK) as a Candidate in Fish

Despite the growing data confirming NO as a key mediator in the cardiac response of fish to O_2_ and temperature stress, further research is needed to discover the complete molecular networks that are orchestrated by this gasotransmitter. A point of attention is the role played by kinases. Largely expressed in cardiac cells and strategically located within the intracellular signalling pathways, these enzymes control protein function and thus a large number of cell activities. In mammals, the importance of the cardiac “kinome” is well assessed (for details, see [142]). Some of these enzymes are crucial for the myocardial response to stress. Examples are the kinases belonging to the RISK and SAFE cascades, of which the role is fundamental for protecting the myocardium against the challenges induced by ischemia/reperfusion [143]. In fish, the cardiac kinome is currently understudied. However, based on the available data, including those above summarized, and on the information in mammals, the contribution of critical kinases to shaping the adaptive response to stress is easy to hypothesize. Together with PKA, PKG, PI3K, Akt, and others, the AMPK represents an interesting candidate. It is a sensitive serine–threonine kinase composed of three subunits (a 63 kDa catalytic α-subunit, a 43 kDa regulatory β-subunit, and a 38 kDa γ-subunit), encoded by different genes, differently expressed in cells and tissues. Its major role is to maintain cellular homeostasis in relation to energy balance [144,145]. High AMP levels, generated when ATP consumption exceeds production, activate the kinase, and this results in a general shift from anabolism to catabolism, a modulation of gene and protein expression, a post-translational modification of metabolic enzymes, and mitochondrial biogenesis [146,147]. The AMPK activity also relates with metabolic redox and oxidative equilibria, although conclusive cause-effect models are still ongoing. It was suggested that the kinase is either inactivated by oxidative stress [148] or stimulated by the exposure to H_2_O_2_ and NO [149]. It is out of our purposes to provide a detailed description of this rich molecular network, and the reader is invited to refer to many excellent and comprehensive reviews for further information.

In the context of this review, an aspect that needs to be highlighted is the relationship between the AMPK and the nitrergic system. The kinase is located upstream of NOS enzymes. In mammalian cultured cardiomyocytes, the AMPK activates eNOS and nNOS by phosphorylation, thus contributing to NO production [150]. At the same time, an AMPK-dependent pathway activates Akt, thus indirectly modulating that in cardiomyocytes is involved in eNOS phosphorylation [151].

In the heart, the relationship between AMPK and the nitrergic system is of importance, particularly under stress. The AMPK is activated under ischemia-reperfusion, increased workload, and glucose uptake impairment, all conditions calling for a reduced O_2_ and ATP, and a perturbation of the oxidative/nitrosative equilibrium [152,153,154]. Notably, the AMPK itself is regulated by NO that acts as an endogenous activator of the kinase. Under normal condition, NO stimulates the AMPK via an sGC/cGMP/Ca^2+^/CaMKβ pathway, while under stress the kinase is activated by the overproduced peroxynitrite via a PKC/LKB1 cascade [149]. The significance of this positive feedback between the AMPK and the nitrergic system, as well as the mechanisms of reciprocal control, is still an issue of active debate in mammals.

The fundamental importance of the AMPK in cell homeostasis is unequivocally confirmed by its presence throughout the evolution of living organisms, from amoebozoa to mammals, and even in plants [155,156]. In teleost fish, consistent with the event of whole genome duplication [157], the AMPK subunits encoding genes are expanded, so that, for example in the zebrafish, 10 AMPK subunits are present [155,156]. This amplifies the functions of the enzyme and complicates the molecular studies performed with experimental schemes and tools (i.e., antibodies) generated for mammalian studies.

So far, the biological role of the enzyme has been explored in several teleost species in relation to different challenges. Molecular and functional data obtained in the hypoxia/anoxia-resistant crucian carp and goldfish [158,159] suggest that the kinase is sensitive to O_2_ reduction in a tissues-specific manner. In the goldfish exposed to 12 h of severe hypoxia, the AMPK is activated in the liver and presumably not in other tissues including heart, brain, and gills [159]. At the moment, it is unclear whether the absence of enzyme activation is one of the physiological compensatory mechanisms that prevent energy decline under low O_2_, such as regional blood shunts and/or activations of alternative metabolic routes able to sustain cell bioenergetics [60]. At the same time, the available data may be influenced by the experimental context. For example, in goldfish, a short time of exposure and a partial O_2_ availability may be not enough to induce enzyme activation [159]. Consistent with this, in the crucian carp, a prolonged exposure (up to 7 days) to severe anoxia, but not hypoxia, is accompanied by an increased activation (i.e., phosphorylation) of the AMPK in the heart. This suggests that the kinase may be quiescent until complete anoxia is achieved [160]. In addition, this event is reversible, since the levels of the phosphorylated enzyme return to pre-anoxic levels after reoxygenation, suggesting an adaptive role [158]. This may be advantageous for hypoxia-tolerant species, since it allows increasing hypoxia tolerance before the AMPK-mediated metabolic adjustments is recruited. In this way, protein synthesis and other AMPK downregulated anabolic pathways continue to function under hypoxia along with the ability to preferentially shunt blood flow to at-risk organs (e.g., brain and heart) [160].

In line with the role as a multistressor-dependent kinase, in fish, the AMPK function is related to temperature challenges, although the role of the kinase is still unclear. In salmonids (*O. mykiss* and *O. kisutch*), the AMPK phosphorylation correlates with optimal temperature for the aerobic scope, measured in terms of the maximum HR [161]. Recent observations on the heart of the olive flounder, *Paralichthys olivaceus*, show that cold stress activates the AMPK together with its upstream modulators (LKB1 and CAMKK) and downstream targets (SITR1, FOXO1A, and TFAM), and this positively affects the fish adaptive response to cold [162]. However, Nilsson and collaborators [163] reported no changes in the AMPK phosphorylation in the heart of crucian carp acclimated to temperatures (4, 10, and 20 °C), which are naturally experienced by the species, suggesting that the kinase remains quiescent within the adaptive thermal range of the animal.

To the best of our knowledge, the interplay between the AMPK and the nitrergic system in the fish heart in relation to stress, either single or multiple, has received scarce, or even null, attention. However, the data above illustrated clearly indicate that both are present and active in the fish heart and are involved in the adaptive response to O_2_ and temperature changes. The possibility that the extreme cardiac flexibility of several fish species, as in the case of cyprinids, may take advantage of signalling pathways that converge on both the NOS/NO system and the AMPK is intriguing and deserves attention.

## 4. Conclusions

It appears that, despite the increasing effort in research, at the moment a unifying picture on the role of the NOS/NO system in the modulation of the cardiac response of the fish heart to stress cannot be drawn because of important factors. One is the different degree of adaptation that characterizes fish species and that moves from those strictly intolerant to those that are highly permissive to stress. Another point is the cardiac architecture characterized by two myocardial arrangements (i.e., a compact and a spongy myocardium), differently expressed in the ventricle of various fish species. Lastly, but not for importance, is the experimental approach carried out at different levels of biological organization from molecular investigations on cells and tissues to physio-pharmacological analyses, either on part (e.g., ventricular strips and rings) or whole (e.g., isolated and perfused preparations) organ, and with different stress administration (e.g., acute or chronic; medium or extreme).

Further explorations are welcome to better describe the nitrergic modulation of fish heart in relation to challenges as O_2_ and temperature variations. Information may contribute to enrich basic knowledge, and this is crucial since it represents an irreplaceable step for all scientific progress. It may also provide a better understanding of the evolutionary history of the mechanisms that protect the heart of non-mammalian models of tolerance, while they are less efficient in mammals, as in the case of the fragile O_2_ deprived ischemic mammalian heart. At the same time, it is important for adaptive and conservative physiology. In the presence of rapid environmental changes, with a growing global temperature rise and a decrease of water O_2_ availability, the mechanisms of adaptation may be over-stressed. The discovery of the mechanisms for compensation which rely on the nitrergic system and its molecular partners may be of value also for supporting actions and strategies of environmental benefit.
